# A quantitative analysis of lymphatic vessels in human breast cancer, based on LYVE-1 immunoreactivity

**DOI:** 10.1038/sj.bjc.6602844

**Published:** 2005-10-25

**Authors:** T Kato, R Prevo, G Steers, H Roberts, R D Leek, T Kimura, S Kameoka, T Nishikawa, M Kobayashi, D G Jackson, A L Harris, K C Gatter, F Pezzella

**Affiliations:** 1 Department of Surgery II, School of Medicine, Tokyo Women's Medical University, 8-1 Kawadacho, Shinjuku-ku, Tokyo 162-8666, Japan; 2 Cancer Research UK Tumor Pathology Group, Nuffield Department of Clinical Laboratory Sciences, University of Oxford, John Radcliffe Hospital, Headington, Oxford OX3 9DU, UK; 3 MRC Human Immunology Unit, Weatherall Institute of Molecular Medicine, John Radcliffe Hospital, Headington, Oxford OX3 9DS, UK; 4 Department of Surgical Pathology, School of Medicine, Tokyo Women's Medical University, 8-1 Kawadacho, Shinjuku-ku, Tokyo 162-8666, Japan; 5 Department of Pathology, School of Medicine, Tokyo Women's Medical University, 8-1 Kawadacho, Shinjuku-ku, Tokyo 162-8666, Japan; 6 Cancer Research UK Molecular Oncology Laboratory, Institute of Molecular Medicine, John Radcliffe Hospital, Headington, Oxford OX3 9DU, UK

**Keywords:** breast cancer, lymphangiogenesis, lymphatic microvessel density, lymphatic vessel invasion, lymphatic tumour dissemination, LYVE-1

## Abstract

This study was undertaken to determine the highly sensitive method for detecting tumour lymphatic vessels in all the fields of each slide (LV), lymphatic microvessel density (LMVD) and lymphatic vessel invasion (LVI) and to compare them with other prognostic parameters using immunohistochemical staining with polyclonal (PCAB) and monoclonal antibodies (MCAB) to the lymphatic vessel endothelial hyaluronan receptor-1 (LYVE-1), and the pan-endothelial marker factorVIII in a series of 67 human breast cancers. In all LYVE-1-stained sections, LV (some of which contained red blood cells) were frequently found localised in extralobular stroma, dermis, connective tissue stroma and adjacent to artery and vein, but were rare within the intralobular stroma or the tumour body (3/67 cases) or areas of widespread invasion. In contrast small blood vessels were observed in intra- and extralobular stroma in the factor VIII-stained sections. Quantitation of vessel numbers revealed that LYVE-1/PCAB detected a significantly larger number of LV than either H&E or LYVE-1/MCAB (*P*<0.0001). LYVE-1/PCAB detected LVI in 25/67 cases (37.3%) and their presence was significantly associated with both lymph node metastasis (*χ*^2^=4.698, *P*=0.0248) and unfavourable overall survival (OS) (*P*=0.0453), while not relapse- free survival (RFS) (*P*=0.2948). LMVD had no influence for RFS and OS (*P*=0.4879, *P*=0.1463, respectively). Our study demonstrates that immunohistochemistry with LYVE-1/PCAB is a highly sensitive method for detecting tumour LV/LVI in breast cancer and LVI is a useful prognostic indicator for lymphatic tumour dissemination.

The common pathways of cancer cell dissemination are *via* the lymphatics and the bloodstream. It has been well established that angiogenesis is necessary for tumour growth and haematogenous metastasis (Weidner *et al*, 1991; Horak *et al*, 1992; Fox *et al*, 1995; Kato *et al*, 2001). However, the role of the lymphatic system is less understood. Some investigators have concluded that it is impractical to distinguish between the blood and lymphatic vessel systems as independent routes of tumour dissemination because they are so interrelated (Fisher and Fisher, 1966). Our previous studies challenge this by demonstrating that differences between blood vessel invasion (BVI) and lymphatic vessel invasion (LVI) in breast cancer can be distinguished by using antibodies against factor VIII related antigen (Kato *et al*, 2002, 2003). However, the distinction between lymphatic vessels and blood vessels was sometimes difficult to determine and hence arbitrary. As yet there have been no specific immunohistochemical markers available that allow the identification of lymphatic vessels. Recent studies have proposed a number of potential lymphatic-specific markers, including the lymphatic vessel endothelial hyaluronan receptor-1 (LYVE-1) (Banerji *et al*, 1999; Jackson, 2004), Prox-1 (Wigle and Oliver, 1999), podoplanin (Breiteneder-Geleff *et al*, 1997), and vascular endothelial growth factor receptor-3 (VEGFR-3) (Kukk *et al*, 1996; Lymboussaki *et al*, 1999; Jackson, 2001) and D2-40 (Kahn *et al*, 2002).

There has been debate in the literature as to whether lymphatic vessels exist within tumours (Leu *et al*, 2000; Nathanson *et al*, 2000; Padera *et al*, 2000; Birner *et al*, 2001; Mandriota *et al*, 2001; Schoppmann *et al*, 2001; Skobe *et al*, 2001; Stacker *et al*, 2001; Beasley *et al*, 2002; Dadras *et al*, 2003; Hall *et al*, 2003; Maula *et al*, 2003; Straume *et al*, 2003; Williams *et al*, 2003); whether tumours induce lymphangiogenesis (Leu *et al*, 2000; Nathanson *et al*, 2000; Sleeman, 2000; Mandriota *et al*, 2001; Schoppmann *et al*, 2001; Skobe *et al*, 2001; Stacker *et al*, 2001; Beasley *et al*, 2002; Williams *et al*, 2003); whether lymphangiogenesis or dilated pre-existing lymphatic vessels increase the probability of lymphatic tumour dissemination (Leu *et al*, 2000; Padera *et al*, 2000; Sleeman, 2000; Dadras *et al*, 2003; Straume *et al*, 2003; Bono *et al*, 2004); and whether cancer cells require active intravasation (Hartveit, 1990; Gunningham *et al*, 2000; Kinoshita *et al*, 2001; Schoppmann *et al*, 2001).

In this study, we used both polyclonal and monoclonal antibodies to the lymphatic-specific marker LYVE-1, a homologue of the CD44 hyaluronan receptor (Banerji *et al*, 1999; Jackson, 2003; Jackson, 2004) to identify lymphatic vessels in breast tumours. In parallel, we also looked at haematoxylin and eosin (H&E) and factor VIII-related antigen staining (FVIII). We determined the presence and distribution of lymphatic vessels and examined the relationships of LVI and lymph-node metastases in a retrospective series of 67 human breast carcinomas.

## PATIENTS AND METHODS

### Tumour collection

In all, 67 consecutive unselected patients who underwent breast cancer surgery at the Tokyo Women’s Medical University Hospital between January 1991 and December 1991 were included in the present study. To be included, the patients (all female) had to have primary, unilateral, breast cancer and no other malignancy. Clinical and pathological data are listed in Table 1. Estrogen and progesterone receptor (ER and PR) content were determined biochemically using the dextran-coated charcoal (DCC) method. Tumours were classified as estrogen receptor or progesterone receptor-positive if the content exceeded 5 fmol *μ*g^−1^.

### Immunocytochemical techniques

Serial sections were prepared from representative formalin-fixed and paraffin-embedded tissue blocks from this series of breast cancer. Tissue samples of 5 *μ*m thick sections stained with haematoxylin and eosin (H&E) were assessed histopathologically and were used to select the maximal area of all the cut surfaces of the tumour that included the invasive components. Immunostains for FVIII-related antigen were performed on paraffin sections using the streptavidin-biotin-immunoperoxidase method as previously described (Kato *et al*, 2002, 2003). Briefly, formalin-fixed, paraffin-embedded sections were dewaxed in 100% Citroclear, rehydrated through graded 100% industrial methylated spirit (IMS) series, and immunostaining was performed using a polyclonal antibody (von Willebrand factor, Dako, Copenhagen, Denmark) applied at 1 : 200 for 1 h at room temperature. Technical details of the polyclonal and monoclonal LYVE-1 staining are outlined in Tables 2 and 3. A normal human tonsil served as a positive control. Rabbit polyclonal antibody and mouse monoclonal antibody to LYVE-1 (LYVE-1/PCAB and LYVE-1/MCAB) were generated as described previously (Banerji *et al*, 1999; Cao *et al*, 2004).

### Assessment of lymphatic vessels

#### H&E staining

We defined as lymphatics those vessels lined by flattened endothelial cells, in the presence or absence of lymphocytes and absence of erythrocytes, in the stroma or adjacent to arteries and veins.

#### LYVE-1/PCAB. staining *and* LYVE-1/MCAB staining

**Figure 1:**
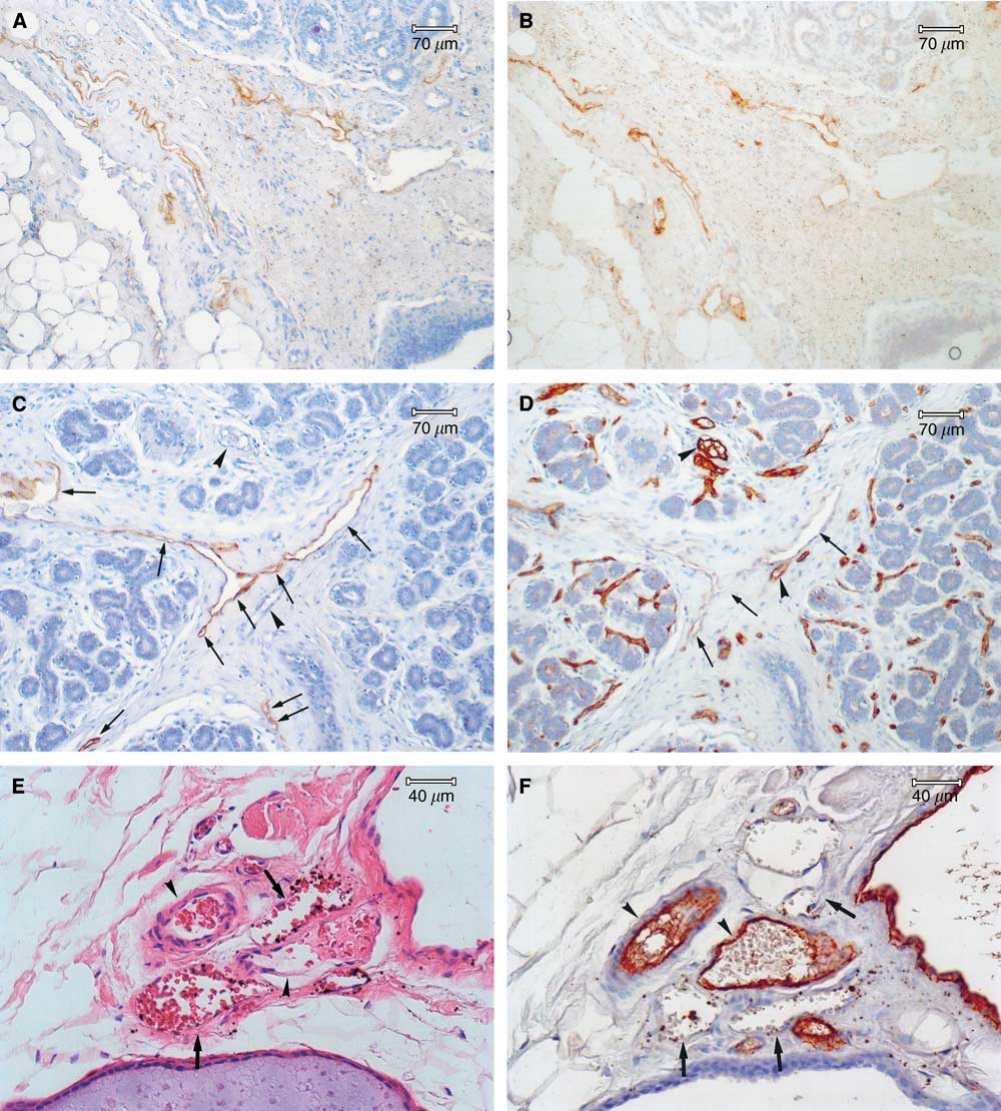

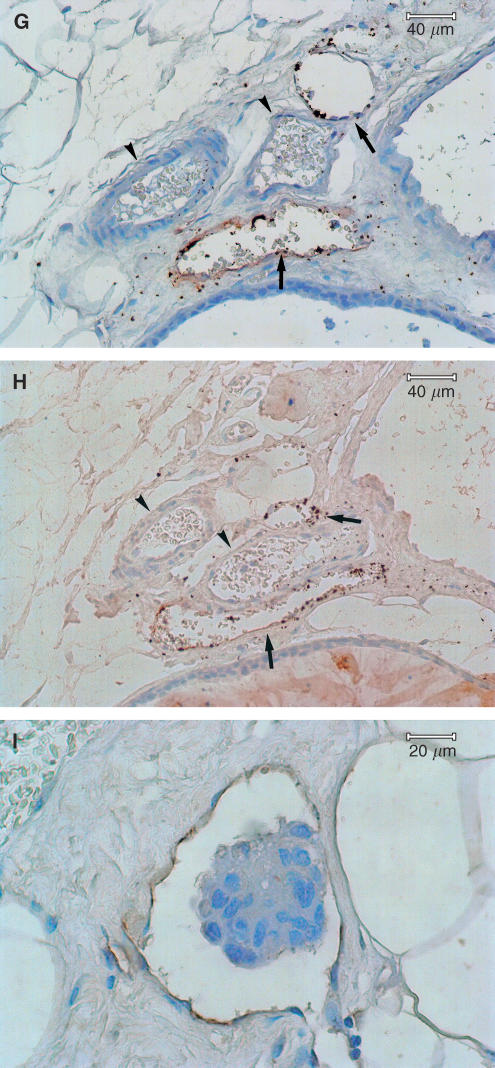
(**A**) Several LYVE-1/PCAB-positive lymphatic vessels are present in the connective tissue stroma (**A**: LYVE-1/PCAB staining, haematoxylin counter stain). (**B**) The monoclonal anti LYVE-1 antibodies (LYVE-1/PCAB) yielded specific and consistent staining of endothelial cells in the lymphatic vessels (**B**: LYVE-1/MCAB staining, haematoxylin counter stain). (**C**) The arrows indicate lymphatic vessels, which are detected by LYVE-1/PCAB staining. We found nine lymphatic vessels in this field. Lymphatics are frequently found in extralobular stroma, but rarely seen in intralobular stroma. Blood vessels (arrowheads) are not stained by the staining (**C**: LYVE-1/PCAB staining, haematoxylin counter stain). (**D**) The arrowheads show blood vessels, which are stained by factor VIII related antigen staining. They are found in both intra- and extralobular stroma. The intensity of endothelial cells in the lymphatic vessels (arrows) in factor VIII related antigen-stained section is very faint, discontinuous and inconsistent (**D**: factor VIII-related antigen staining, haematoxylin counter stain). (**E**, **F**, **G** and **H**) Serial sections were prepared for 4 kinds of staining. Some lymphatic vessels (arrows), which contained red blood cells, were observed in haematoxylin and eosin (H&E), factor VIII related antigen, LYVE-1/PCAB and LYVE-1/MCAB-stained sections. Arrows indicate lymphatic vessels and arrowheads show blood vessels (**E**: H&E staining; **F**: factor VIII related antigen staining; **G**: LYVE-1/PCAB staining; **H**: LYVE-1/MCAB staining, haematoxylin counter stain). (**I**) A lymphatic vessel with floating tumour cells (LVI) was found localised in the connective tissue stroma (**I**: LYVE-1/PCAB staining, haematoxylin counter stain).

Positive vessels were scored as lymphatic vessels. Staining intensity was assessed as follows; strong staining; moderate staining; weak staining (Figure 1A and B).

### Counting of lymphatic vessels and determination of lymphatic microvessel density (LMVD) and blood microvessel density (BMVD)

Both the number and intensity of staining of the lymphatic vessels were evaluated. The intensity of staining and level of tissue damage were expressed as weak, moderate and strong. We defined as a lymphatic vessel the vessel, which have endothelium with immunopositivity and a vascular lumen. Mean lymphatic vessel count was determined by averaging the number of total lymphatic vessels in all the fields of each slide, including within the tumour or at the periphery of the tumour, at × 100 or × 200 magnification. Single brown-stained endothelial cells with a lumen were counted as individual lymphatic vessels, as shown in Figure 1C. The three most vascularised areas (‘hot spots’) were selected at low power magnification (× 40) and LMVD and BMVD were then determined by counting all LYVE-1/PCAB-immunostained or factor VIII related antigen stained vessels at × 200 magnification. When the average number was higher than the median number of LYVE-1/PCAB or FVIII related antigen positive vessels, the cancer was considered to have a high LMVD or BMVD, otherwise a low LMVD or BMVD.

### Statistical analysis

Statistical analysis of the data was performed with the Survival Tools for Statview-J 5.0. package (Abacus Concepts, Berkeley, CA, USA). For comparison of number of lymphatic vessel assessed by the three different staining methods, for association of LMVD and clinical or pathologic parameters and for the association of LVI and lymph-node status, Kruskal–Wallis test, Mann–Whitney *U*-test and *χ*^2^ test were used. The association of the numbers of lymphatic vessels in the LYVE-1/PCAB and those in LYVE-1/MCAB stained sections was assessed by Pearson’s correlation coefficient. We examined the univariate relationships between prognostic indicators and relapse-free survival (RFS) and overall survival (OS) by fitting Kaplan–Meier survival curves (Kaplan and Meier, 1958) to various levels of the prognostic indicators.

## RESULTS

Both the polyclonal and monoclonal anti LYVE-1 antibodies yielded specific and consistent staining of endothelial cells in the lymphatic vessels (Figure 1A and B). Many lymphatic vessels were frequently detected in dermis, connective tissue stroma (Figure 1A and B), retro-mammary tissue, adjacent to artery and vein and extralobular stroma (Figure 1C). However, lymphatic vessels were rarely seen in intralobular stroma (Figure 1C), intra-tumour tissue, areas of necrosis, adipose tissue (Figure 1A and B) and muscle. In contrast, in the FVIII-stained sections small blood vessels were observed in both intra- and extralobular stroma (Figure 1D). In addition to those findings many lymphatic vessels, which contained red blood cells were observed in H&E, FVIII staining, LYVE-1/PCAB and LYVE-1/MCAB-stained sections. (Figure 1E–H). It was difficult to distinguish between lymphatic vessels and blood vessels by the finding of the presence or absence of erythrocytes in the lumen of vessels detected by H&E staining alone.

The mean and median (range) number of all lymphatic vessels is shown in Table 4. The total and the mean number of LYVE-1/PCAB-immunostained lymphatic vessels were higher than that of the H&E and LYVE-1/MCAB- stained lymphatic vessels. (*P*<0.0001). Strong significant correlation was between the LYVE-1/PCAB-immunostained lymphatic vessels and LYVE-1/MCAB-immunostained lymphatic vessels (Pearson’s correlation coefficient=0.815, *P*<0.0001). Median LMVD was 6.1 microvessels mm^−2^ (range 0–17.9 vessels). A strong significant correlation was found between LMVD and LYVE-1/PCAB-immunostained lymphatic vessels (Pearson’s correlation coefficient=0.718, *P*<0.0001). There was no significant correlation between the LMVD and BMVD (Pearson’s correlation coefficient=0.021, *P*=0.8710). An inverse correlation was seen between histological grading and LMVD (*P*=0.0434), while histological grading or menopausal status trended with the number of lymphatic vessels (*P*=0.0712) or LMVD (*P*=0.0944). There was no significant correlation between clinical tumour size, lymph-node status, LVI, or estrogen receptor and LMVD or the mean number of lymphatic vessels (Table 5). LVI was detected by H&E, LYVE-1/PCAB and LYVE-1/MCAB staining in 23/67 cases (34.3%), 25/67 cases (37.3%) and 20/67 cases (29.9%), respectively. The lymph-node status or LVI detected by H&E, LYVE-1/PCAB and LYVE-1/MCAB was not associated with the mean number of lymphatic vessels (*P*=0.6413, *P*=0.8339, *P*=0.8884 or *P*=0.7412, *P*=0.5759; *P*=0.8075, respectively), but LVI detected by LYVE-1/PCAB was significantly associated with lymph-node status (*χ*^2^=4.698, *P*=0.0248, Table 6). A significant difference in OS was found between patients with LVI or without LVI (*P*=0.0453), while no significant difference in RFS (*P*=0.2948). However, LMVD had no influence for OS and RFS (*P*=0.4879, *P*=0.1463, respectively).

## DISCUSSION

This manuscript has focused on the utility of different LYVE-1 antibodies as routine markers for detecting and quantitating lymphatic vessels in breast cancer. Our results confirm that both LYVE-1 polyclonal and monoclonal antibodies distinguish efficiently between lymphatic and blood vessels in pathological specimens. However, due to the greater dependence of LYVE-1 monoclonal antibodies on tissue fixation and antigen retrieval methods that result in partial antigen destruction, we have found that LYVE-1 polyclonal antibodies are suited to routine immunohistochemical staining applications. Furthermore, using immunohistochemical staining with LYVE-1 polyclonal antibodies we have shown that lymphatic invasion is positively associated with lymph node involvement and unfavorable OS.

Quantitation of tumour lymphatic vessels for the purpose of tumour staging has for decades been problematic. Although morphology can sometimes distinguish lymphatic vessels from blood vessels by the frequent absence of a basement membrane and lack of erythrocytes in the latter, neither is a reliable method for routine use. These considerations have hampered the reliable identification of tumour lymphatic vessels in routine histopathology. More recently, however, the development of specific markers such as the lymphatic hyaluronan receptor LYVE-1, the subject of this manuscript, has allowed many new experimental studies of tumour lymphatics to be initiated. To date, the majority of these studies have employed LYVE-1 polyclonal antibodies, requiring microwave treatment or pressure cooking for antigen retrieval (Banerji *et al*, 1999; Beasley *et al*, 2002; Williams *et al*, 2003). In this present manuscript, we found that the intensity of many endothelial cells in the lymphatic vessels of either microwave or pressure cooking treatment stained with LYVE-1/PCAB was similar to that seen with protease retrieved tissue sections. However, some breast tissue sections in the former treatment were damaged, so it was difficult to observe the lymphatic vessels of all the fields in each slide. These results confirm that proteolytic enzyme treatment for LYVE-1/PCAB staining as well as for FVIII staining is more useful than either microwave or pressure cooking treatment in the human breast tissue. The LYVE-1/MCAB requires the use of microwave antigen retrieval to produce good staining without background. The staining of lymphatic vessels in LYVE-1/MCAB–stained sections was occasionally more intense than that in LYVE-1/PCAB–stained sections (Figure 1A and B). However, because of the use of microwave antigen retrieval, section quality is often low so that the estimation of the total number of lymphatic vessels is less reliable than with LYVE-1/PCAB staining.

Previous studies have reported that all blood vessels that contain erythrocytes are negative for LYVE-1 supporting its specificity for lymphatics (Banerji *et al*, 1999; Williams *et al*, 2003). However, in this study, we observed some LYVE-1-positive vessels containing erythrocytes in both LYVE-1/PCAB and LYVE-1/MCAB-stained sections (Figure 1E–H). A prominent function of the lymphatic system is the provision of fluid drainage of lymphocytes, protein, colloid and foreign antigens from the tissues to the peripheral lymph nodes. Moreover lymphatic vessels act as a conduit for both migrating inflammatory cells and possibly erythrocytes from inflammatory tissue and parenchyma or stroma with haemorrhage. Hartveit has described tumour cells in the periductal lymphatic spaces being washed with the tide of interstitial fluid into the lymphatic network and into the lymphatic vessels (Hartveit, 1990). Therefore lymphatic vessels containing erythrocytes may well be observed from time to time especially in tumours. Based on similar findings in tumours by Padera the specificity of LYVE-1 for lymphatics has recently been questioned. This can be explained either by the presence of haemovascular-lymphovascular shunts (Clarijs *et al*, 2001; Abtahian *et al*, 2003), by leakiness of newly proliferating tumour blood capillaries that is high degree of fenestration, or rupture giving rise to RBC that enter the lymphatic vessels. It is possible that there are more of these in tumours further explaining our findings of erythrocytes in LYVE-1 positive vessels.

Several recent studies in animal models have reported that lymphatic vessels are frequently observed in the peripheral rim of the tumour, but not in the body of the tumour itself (Leu *et al*, 2000; Padera *et al*, 2000). However, others demonstrated the existence of intratumoural lymphatic vessels, in xenotransplanted breast tumours and fibrosarcomas in mice and in human head and neck cancers respectively (Skobe *et al*, 2001; Stacker *et al*, 2001; Beasley *et al*, 2002; Jackson, 2004). Most studies in human breast cancer described a similar peritumoural distribution (Nathanson *et al*, 2000; Schoppmann *et al*, 2001; Williams *et al*, 2003; Bono *et al*, 2004) which is largely supported by the current study which shows that lymphatic vessels are frequently found in the extralobular stroma and connective tissue stroma (Figure 1A–C) but are rarely seen in the intralobular stroma and within the tumour itself. The reasons for this selective localisation of lymphatic vessels in tumours is unknown but one possibility is that they are collapsed in expanding tumours because of the high interstitial pressure. That would suggest that they are present, but difficult to detect because of their flattened morphology. In any case ‘collapsed’ is a subjective interpretation of appearance in histological sections. Even presumably fully functional lymphatic vessel in normal tissue can look ‘collapsed’. The growth of tumour lymphatics is likely to be limited by availability of lymphangiogenic growth factors, related to infiltration by tumour associated macrophages (Williams *et al*, 2003), physical barriers to intratumoural lymphatic vessel growth (Mandriota *et al*, 2001) or inhibitory mechanisms as yet undefined, but perhaps similar to those that appear to prevent lymphatic vessel growth in the cornea (Cursiefen *et al*, 2002). Those lymphatic vessels which are present at the tumour periphery are considered to be pre-existing lymphatics rather than those induced by tumour lymphangiogenetic factors (Leu *et al*, 2000).

Recent studies have demonstrated that VEGFR-3 or D2-40 immunostained microvessels was associated with either node metastases (Nathanson *et al*, 2000) or BMVD (Choi *et al*, 2005) and a high peritumoural lymphatic vessel density is associated with a poor outcome in human breast cancer (Bono *et al*, 2004). On the other hand, other researchers reported that there was no significant correlation between LMVD and BMVD (Schoppmann *et al*, 2001; Bono *et al*, 2004), tumour size, histological grading or nodal status (Williams *et al*, 2003) and no significant difference between high lymphatic vessel density and low one for RFS and OS (Schoppmann *et al*, 2004). The results of our study suggest that an inverse correlation was seen between LMVD and histological grading, while there was no significant correlation between LMVD and lymph-node status or LVI and LMVD was not associated with a poor outcome. As there was no significant correlation between LMVD and BMVD, the genesis of lymphatic vessels might be different from that of blood vessels (Schoppmann *et al*, 2001). It is tempting to speculate that as lymphatics in tumour with increased aggressiveness could be excluded and destroyed by cancer (Williams *et al*, 2003), LMVD has no influence for lymphatic tumour dissemination.

The current study has used LYVE-1 staining to increase the accuracy and rate of detection of LVI since using this method makes it easy to distinguish from BVI (Figure 1I). In recent human studies the rate of LVI fell to within the range of 13.3 and 53.3% (Schoppmann *et al*, 2001; Williams *et al*, 2003), while the rate of LVI detected by LYVE-I/PCAB staining was seen in 25/67 cases (37.3%) in this study, and the potential importance of measuring the LVI is that it has been strongly associated with the presence of lymph-node metastases and unfavorable OS in human breast cancer (Schoppmann *et al*, 2004). The results in this present study support that suggestion.

## Figures and Tables

**Table 1:** Clinicopathologic characteristics of 67 patients

**Characteristics**	**No. of patients (%)**
Patients enrolled	67
*Age (years)*	
Median	49
Range	30–86
	
*Menopausal status*	
Pre	36 (53.7)
Post	31 (46.3)
	
*Clinical tumor size, T*	
T1	26 (38.8)
T2	34 (50.7)
T3	7 (10.5)
	
*Lymph-node status*	
Negative	43 (64.2)
Positive	20 (29.9)
Unknown	4 (5.9)
	
*ER*	
Negative	29 (43.3)
Positive	35 (52.2)
Unknown	3 (4.5)
	
*PR*	
Negative	38 (56.7)
Positive	26 (38.8)
Unknown	3 (4.5)
	
*Histological classification*	
Noninvasive ductal carcinoma	5 (7.5)
Infiltrating ductal carcinoma	57 (85.0)
Infiltrating lobular carcinoma	1 (1.5)
Others	4 (6.0)

ER=estrogen receptor; PR=progesteron receptor.

**Table 2:** The method for polyclonal antibody against LYVE antigen

1	Deparaffinise sections in 100% citroclear for 10 min
2	Rehydrate through graded 100% industrial methlated spirits (IMS) series for 5 min
3	Predigest with 0.1% Protease for 5 min
4	Abolish endogenous peroxidase activity with 3% hydrogen peroxide for 20 min
5	Leave slides to tap water for 5 min
6	Wash in phosphorate-buffered saline (PBS, pH 7.0)
7	Suppress nonspecific background staining with 5% normal human serum for 15 min
8	Apply primary antibody (1 : 600 diluted LYVE-I polyclonal antibody in PBS) for 1 h at room temperature
9	Wash in PBS for 5 min
10	Apply secondary antibody (DAKO anti-rabbit envision HRP polymer) for 30 min at room temperature
11	Wash in PBS for 5 min
12	Apply 0.05% 3,3′-diaminobenzidine tetrahydrochloride (DAB) substrate provided in envision kit for 4 min
13	Wash in distilled water
14	Counter-staining by hematoxylin
15	Aquamount

LYVE: lymphatic vessel endothelial hyaluronan receptor-1.

**Table 3:** The method for monoclonal antibody against LYVE antigen

1	Deparaffinise sections in 100% citroclear for 10 min
2	Rehydrate through graded 100% industrial methlated spirits (IMS) series for 5 min
3	Antigen retrieve; microwave in Dako target antigen retrieval buffer diluted 1 : 10 at 95–100°C for 40 min
4	Wash in water, rinse distilled water, then transfer tris-buffered saline (TBS)
5	Abolish endogenous peroxidase activity with peroxidase block from Dako envision kit for 5 min
6	Wash in TBS for 5 min
7	Apply primary antibody (LYVE-I monoclonal antibody diluted 1 : 2 with 0.1% bovine serum albumin in TBS) at 4°C over night
8	Wash in TBS for 5 min
9	Apply secondary antibody (DAKO anti-mouse envision HRP polymer) for 30 min at room temperature
10	Wash in TBS for 5 min
11	Apply 0.05% 3,3′-diaminobenzidine tetrahydrochloride (DAB) substrate provided in envision kit for 5 min
12	Wash in TBS, rinse distilled water
13	Counter-staining by hematoxylin
14	Aquamount

LYVE=lymphatic vessel endothelial hyaluronan receptor-1.

**Table 4:** Comparison of the three methods for detection of lymphatic vessels

**Methods of staining**	**H&E**	**LYVE-1/PCAB**	**LYVE-1/MCAB**	***P*-value**
*Number of lymphatic vessels*				
Total number of 67 cases	4274	17334	10919	
Mean±s.d.	63.8±64.4	258.7±219.1	163.0±155.7	<0.0001
Median	45	204	109	
Range	4–372	0–828	2–575	
				
*Level of expression (No. of patients)*				
Weak		19 (28.4%)	25 (37.3%)	
Moderate		19 (28.4%)	25 (37.3%)	
Strong		29 (43.2%)	17 (25.4%)	
				
*Level of tissue damage (No. of patients)*				
Weak		59 (88.1%)	5 (7.5%)	
Moderate		8 (11.9%)	48 (71.6%)	
Strong		0	14 (20.9%)	

H&E=hematoxylin and eosin; LYVE-1=lymphatic vessel endothelial hyaluronan receptor-1; PCAB=polyclonal antibody; MCAB=monoclonal antibody.

**Table 5:** Association of LMVD and other clinical and pathologic parameters

	**No. of patients (%)**	**Mean LMVD**	***P*-value**	**Mean LV**	***P*-value**
Patients enrolled	67	6.3±4.2		258.7±219.1	
*Clinical tumor size, T*			0.3061		0.5812
T1	26 (38.8)	7.3±4.6		277.1±219.0	
T2	34 (50.7)	5.8±3.9		258.4±228.6	
T3	7 (10.5)	4.7±3.5		191.5±184.4	
					
*Histological grading, HG*			0.0434		0.0712
HG1	21 (31.4)	6.5±3.1		287.3±200.1	
HG2	24 (35.8)	7.6±4.8		307.1±252.8	
HG3	22 (32.8)	4.6±3.9		178.5±180.1	
					
*Lymph-node status*			0.8305		0.4764
Negative	43 (64.2)	6.4±4.1		254.2±213.1	
Positive	20 (29.9)	6.3±4.5		242.4±188.9	
Unknown	4 (5.9)	4.8±2.9		342.2±351.2	
					
*Lymphatic vessel invasion*			0.525		0.3088
Negative	42 (62.7)	5.9±3.8		231.1±197.6	
Positive	25 (37.3)	6.8±4.8		305.0±248.4	
					
*Menopausal status*			0.0944		0.1609
Pre	36 (53.7)	6.9±4.1		287.1±205.5	
Post	31 (46.3)	5.5±4.2		225.7±232.9	
					
*Estrogen receptor*			0.691		0.8451
Negative	29 (43.3)	5.8±4.0		257.3±208.8	
Positive	35 (52.2)	6.6±4.4		267.1±235.4	
Unknown	3 (4.5)	7.2±4.6		174.0±140.8	

LMVD=lympatic microvessel density; LV=the number of total lymphatic vessels in all the fields of each slide.

**Table 6:** Association of LVI detected by three kinds of staining and lymph-node status

		**Lymph-node statusc**		
		**Negative**	**Positive**	
**Methods of staining**	**No. of patients**	**No. of patients (%)**	**No. of patients (%)**	***P*-value**
*LVI/H&E*				0.1293
Negative	40	30 (75.0)	10 (25.0)	
Positive	23	13 (56.5)	10 (43.5)	
				
*LVI/LYVE-1/PCAB*				0.0146
Negative	39	31 (79.5)	8 (20.5)	
Positive	24	12 (50.0)	12 (50.0)	
				
*LVI/LYVE-1/MCAB*				0.2457
Negative	44	32 (72.7)	12 (27.3)	
Positive	19	11 (57.9)	8 (42.1)	

LVI=lymphatic vessel invasion; H&E=hematoxylin and eosin; LYVE-1=lymphatic vessel endothelial hyaluronan receptor-1; PCAB=polyclonal antibody; MCAB=monoclonal antibody. Four cases with unknown lymph-node status were excluded.
